# Biological Efficacy of Medicinal Plant Extracts in Preventing Oxidative Damage

**DOI:** 10.1155/2018/7904349

**Published:** 2018-09-13

**Authors:** Jaideep Banerjee, Amitava Das, Mithun Sinha, Sudipta Saha

**Affiliations:** ^1^George Washington University, Washington, USA; ^2^The Ohio State University, Columbus, USA; ^3^Babasaheb Ambedkar University, Lucknow, India

Reactive oxygen species (ROS) and reactive nitrogen species (RNS) are important signaling molecules that maintain cellular homeostasis. Redox imbalance or production of excess amounts of ROS and RNS, however, is either a cause or an important mediator in the pathogenesis and pathophysiology of many diseases. It results in oxidative damage to various biological macromolecules including DNA, lipids, and proteins, thereby altering several signaling pathways that ultimately promote cellular damage and death.

Natural product-based medicines have been used in medical practices for centuries. Naturally derived compounds have fewer reported side effects than allopathic medicine and may be safer to use over a longer period of time. F. Zhu et al. had reported in 2012 in *Plos One* that the active ingredients in combinations of natural products can achieve the same level of potency as synthetic drugs, although they may have to be taken in larger quantities or for a longer period. About 8% of hospital admissions in the United States of America are due to adverse or side effects of synthetic drugs, and approximately 100,000 people each year die due to these toxicities, as reported in *J Appl Pharmaceut Sci* in 2011 by G Philomena. However, toxicity of herbal medicines needs to be seen in context, and although generally considered safe, it can still have side effects.

Many natural compounds and natural product mimics are potential antioxidants that protect against oxidative damage in chronic diseases. Understanding and validating the bioactivities of the natural compounds and the molecular mechanisms are essential for a solid scientific foundation for their clinical use, improvement in their efficacy, and to meet the regulatory challenges. This special issue on the “Biological Efficacy of Medicinal Plant Extracts in Preventing Oxidative Damage” presents a collection of original reports and review articles on the scientific mechanism of action of some novel as well as traditionally used medicinal extracts in preventing oxidative damage-related diseases.

G.-H. Li et al. describe the bioactive constituents and the mechanism of action of Salviae Miltiorrhizae Radix et Rhizoma (SMRR), which is a traditional Chinese medicine and is commonly used for the therapy of cardiac cerebral diseases. The authors discuss the effect of the SMRR extract as well as the purified constituents tanshinone I, tanshinone IIA, and salvianolic acids A and B on the Nrf2 pathway and the resulting antioxidant therapeutic effects on cardiovascular diseases, neurodegenerative diseases, diabetes, nephropathy, inflammation, liver diseases, and lung diseases.

K. C. dos Santos et al. evaluated the effect of the leaves of Yacon (*Smallanthus sonchifolius*) on dysmetabolism and cardiomyopathy in type 1 diabetic rats. Yacon is a native Andean plant that is rich in phenolic compounds, and the treatment increased the activity of the antioxidant enzymes (catalase, superoxide dismutase, and glutathione peroxidase). This was also associated with reduced glycemia, increased insulin concentration, decreased serum triacylglycerol and fatty acid content, and decreased fibrosis and cellular disorganization in the pancreas and cardiac tissue of diabetic animals.

*Carapa guianensis* (Aublet) is a neotropical tree found in the north of South America, Central America, Caribbean, and Sub-Saharan Africa. The seed oil is widely used in Brazilian traditional medicine because of its multiple curative properties against fever and rheumatism and as an anti-inflammatory agent, antibacterial agent, and insect repellant. Authors C. F. Araujo-Lima et al. have evaluated the chemical composition, free-radical scavenging activity, and mutagenic and genotoxicity properties of three *C. guianensis* oils obtained by different extraction methods and have identified the best procedure to extract the oil which makes it safe for use.

Authors D. Guo et al. report that natural *Gracilaria lemaneiformis* sulfated polysaccharide increased the cell viability and restored the cell morphology of human kidney proximal tubular epithelial cells (HK-2) damaged by oxalate. A decrease in released lactate dehydrogenase and an increase in mitochondrial membrane potential were observed. The authors also found that the repair ability of the GLP fractions are closely correlated with the molecular weight of the fractions, with GLP2 exhibiting the strongest repair effect. These results can therefore provide references for inhibiting the formation of kidney stones and developing original antistone polysaccharide drugs.

In the review article by M. A. Mendez-Encinas et al., the authors describe the functional properties and potential application as an antioxidant and anticancer agent of ferulated arabinoxylans, which are polysaccharides obtained from the cell walls of cereal grains. They also discuss the gel-forming characteristic of these polysaccharides, which has characteristics such as high water absorption capacity, stability to pH, temperature, and ionic charges, thus making them an excellent drug delivery system.

J. Meng et al. report a potential use of a traditional Tibetan medicine, *Rheum tanguticum* (*Rt*), for treatment in Alzheimer's disease. *Rt* has anti-inflammatory and antioxidative properties and inhibits the expression and production of inflammatory and oxidative molecules such as IL-1*β*, TNF-*α*, and nitric oxide by microglia. They further found that aloe-emodin and (+)-catechin are responsible for these properties through the secretion of IL-10 from microglia.

The effect of olive leaf extract (OLE) on testicular damage was tested in rats by R. S. Almeer et al. Cisplatin is widely used as an antineoplastic drug for treating various cancers. However, its use is mainly limited by severe toxicity to normal tissues, especially nephrotoxicity, neurotoxicity, and testicular damage. Cisplatin causes disorganization of germinal epithelium and apoptosis. And testicular weights, catalase, serum testosterone, and testicular enzymes are significantly reduced. The authors report that OLE treatment can markedly attenuate both biochemical and histopathological changes and is mediated, at least partly, by inducing the nuclear factor erythroid 2-related factor 2 (Nrf2)/heme oxygenase 1 (HO-1) pathway.

H. A. Ogaly et al., in their manuscript, have investigated the efficacy of *Mentha piperita L.* essential oil (MPEO) against liver fibrosis in rats and have explored this use of MPEO as an antifibrotic treatment for treating chronic liver diseases. Hepatoprotective effects of MPEO were observed as documented by the reduction of liver injury markers and lipid peroxidation (LPO) with ameliorated pathological and fibrotic liver injuries. Furthermore, reduced expression of desmin, *α*-SMA, TGF-*β*1, and SMAD3 proteins indicated that reduced hepatic stellate cell (HSC) activation. MPEO also resulted in downregulation of CCl4-stimulated p53 expression.

Lycopene, which is a potent antioxidant carotenoid, has been evaluated by N. Stojiljkovic et al. in methotrexate-induced kidney damage in rats. Lycopene was administered in two different forms: dissolved in corn oil or encapsulated in nanoliposomes. Application of both forms of lycopene concomitantly with methotrexate was found to be effective against changes in serum urea and creatinine and oxidative damage markers and markedly reversed structural changes of kidney tissue, with the nanoliposome-encapsulated form being more effective for recovery.

Resveratrol (RSV), a natural polyphenol, is known for its potent antioxidant and anticancer effects. Authors B. Yan et al. studied the effect of RSV on the biological properties of activated pancreatic stellate cells that initiate pancreatic fibrosis in chronic pancreatitis. The authors report that RSV downregulates miR-21 expression and induces PTEN expression, resulting in impeded reactive oxygen species induction in PSCs. Collectively, the authors conclude that RSV inhibits invasion and migration of pancreatic cancer cells through suppression of ROS/miR-21-mediated activation and glycolysis in PSCs and thus may serve as a new strategy for clinical prevention or treatment of pancreatic ductal adenocarcinoma.

Taken together, the articles in this special issue contributed by the experts in the fields of oxidative stress biology highlight the increasing importance of investigating the effect of natural products on ameliorating oxidative damage and thus identify safe therapeutic treatments for the plethora of oxidative stress-related diseases.

